# Epigallocatechin-3-gallate suppresses the global interleukin-1beta-induced inflammatory response in human chondrocytes

**DOI:** 10.1186/ar3368

**Published:** 2011-06-17

**Authors:** Nahid Akhtar, Tariq M Haqqi

**Affiliations:** 1 Department of Medicine/Rheumatology, MetroHealth Medical Centre, Case Western Reserve University, 2500 MetroHealth Drive, Cleveland, OH 44109, USA

## Abstract

**Introduction:**

Epigallocatechin-3-gallate (EGCG) is a bioactive polyphenol of green tea and exerts potent anti-inflammatory effects by inhibiting signaling events and gene expression. Interleukin-1beta (IL-1β) is the principal cytokine linked to cartilage degradation in osteoarthritis (OA). The objective of this study was to evaluate the global effect of EGCG on IL-1β-induced expression of proteins associated with OA pathogenesis in human chondrocytes.

**Methods:**

Primary OA chondrocytes were pretreated with EGCG (10 to 100 uM) and then stimulated with IL-1β (5 ng/ml) for 24 hours. Culture supernatants were incubated with cytokine antibody arrays and immunoreactive proteins (80 proteins) were visualized by enhanced chemiluminiscence. Effect of EGCG on IL-1β-induced expression of 18 selected genes was verified by Real time-PCR and effect on IL-6, IL-8 and tumor necrosis factor-alpha (TNF-α) production was determined using specific ELISAs. Western immunoblotting was used to analyze the effect of EGCG on the interleukin-1 receptor-associated kinase 1 (IRAK-1) and TNF receptor-associated factor 6 (TRAF-6) proteins in IL-1β-stimulated chondrocytes. The role of nuclear factor kappa-B (NF-κB) and mitogen activated protein kinases (MAPKs) in the regulation of selected genes and the mechanism involved in EGCG mediated modulation of these genes was determined by using specific inhibitors for NF- κB (MG132) and MAPKs (p38-MAPK, SB202190; JNK-MAPK, SP600125, ERK-MAPK, PD98059).

**Results:**

Out of 80 proteins present on the array, constitutive expression of 14% proteins was altered by EGCG treatment. No significant stimulatory effect was observed on the proteins associated with cartilage anabolic response. Stimulation with IL-1β enhanced the expression of 29 proteins. Expression of all 29 proteins up-regulated by IL-1β was found to be suppressed by EGCG. EGCG also inhibited the expression of the signaling intermediate TRAF-6 at 50 and 100 uM concentrations (*P *< 0.05). Our results identified several new targets of EGCG, including epithelial neutrophil activating peptide-78 (ENA-78), granulocyte macrophage colony stimulation factor (GM-CSF), growth- related oncogene (GRO), GRO-α, IL-6, IL-8, monocyte chemotactic protein-1 (MCP-1), MCP-3, macrophage inflammatory protein-1beta (MIP-1β), granulocyte chemotactic protein-2 (GCP-2), MIP-3alpha, interferon-gamma-inducible protein-10 (IP-10), nucleosome assembly protein-2 (NAP-2) and leukemia inhibitory factor (LIF). The inhibitory effects of EGCG were mainly mediated by inhibiting the activation of NF-κB and c-Jun N-terminal Kinase (JNK)-MAPK in human chondrocytes.

**Conclusions:**

Our results suggest that the potential of EGCG in OA treatment/prevention may be related to its ability to globally suppress the inflammatory response in human chondrocytes. These results identify additional new targets of EGCG and advocate that EGCG may be a potent chondroprotective agent in OA.

## Introduction

Osteoarthritis (OA) is a multifactorial degenerative joint disease which involves articular cartilage matrix destruction and for which there is no cure and no useful treatments to block disease progression. The extracellular matrix of the cartilage is maintained by equilibrium between anabolic and catabolic activities of the chondrocytes - the only cell type present in the cartilage [1,2]. OA essentially reflects an imbalance between matrix anabolic and catabolic processes [2,3]. Multiple pro-inflammatory cytokines such as IL-1β, TNF-α, IL-6 and chemokines (IL-8 and others) are produced by activated chondrocytes in OA [3-6]. IL-8 is a chemoattractant factor involved in synovial inflammation in the joint [4] and IL-6 reportedly plays a contributory role to the OA pathogenesis by increasing the number of inflammatory cells in synovial tissue, stimulating proliferation of chondrocytes, and inducing amplification of IL-1 effects [6]. IL-1β is an inflammatory cytokine and its inhibition has been shown to ameliorate osteoarthritis-like pathology in animal models [7,8]. Further, the role of IL-1β in OA pathogenesis was also been substantiated by studies in IL-1 deficient mice [7,8]. Thus, IL-1β can shift the balance between the biosynthesis and the degradation of extracellular matrix components (via production of matrix metalloproteinases (MMPs), and disintegrin and metalloproteinase with thrombospondin motifs [ADAMTSs], in the cartilage and transform chondrocytes to display the catabolic phenotype seen in OA [3]. Such an imbalance between the anabolism and catabolism of the extracellular matrix is thought to lead to the disruption of cartilage homeostasis and favors degradation, culminating in the loss of joint function [1,9]. The beneficial effects ascribed to drinking green tea (*Camellia sinensis*) are believed to rely on the pharmacological actions of catechins. Green tea is a rich source of catechins and EGCG constitutes up to 63% of total catechins [10]. EGCG has been shown to be 25 to 100 times more potent than vitamins C and E in anti-oxidant activity [11]. Earlier studies have demonstrated the potential chondroprotective effects of EGCG *in vitro *[12-14]. In addition, we have also reported the prevention of inflammatory arthritis by a polyphenol rich extract of green tea in a mouse model of RA [15]. EGCG has been shown to inhibit metalloproteinases [16], nitric oxide [14], cyclooxygenase-2 (COX-2) and production of prostaglandin E_2 _[14], activation of nuclear factor-kappaB (NF-κB) [12], mitogen activated protein kinases (MAPKs) [13] and activator protein-1 (AP-1) [13] in IL-1β stimulated human OA chondrocytes. In the present study, we used antibody based arrays to analyze the global effect of EGCG on IL-1β-induced expression of growth factors, angiogenic factors, cytokines and chemokines in human OA chondrocytes and validated the effect on gene expression of selected proteins by real-time PCR. Our data suggest that EGCG exerts a wide ranging effect on the expression of proteins associated with OA pathogenesis and correlated with the inhibition of NF-κB and MAPKs in human OA chondrocytes. Our results also identify several new targets of EGCG and its unique mechanism of action on these pro-inflammatory genes and suggest the use of EGCG may be of value in the prevention/treatment of inflammation associated with OA.

## Materials and methods

### Cartilage sample and culture of chondrocytes

This protocol to use discarded and de-identified human cartilage was reviewed by the Institutional Review Board (IRB) of the MetroHealth Medical Center, which classified it as exempt from review and that informed consent was not required (IRB09-01330). Human chondrocytes were prepared by enzymatic digestion of cartilage obtained from 15 OA patients (mean age, 60.6 ± 4.7 years) undergoing knee arthroplasty at the MetroHealth Medical Center, Cleveland, OH. OA was diagnosed according to the American College of Rheumatology criteria [17,18]. Specimens that included the full thickness cartilage and subchondral bone were washed with sterile PBS and the macroscopic cartilage degeneration was determined by staining with India ink [19]. Portions of the cartilage with smooth articular surface were used to prepare chondrocytes by the enzymatic digestion with pronase for two hours followed by collagenase digestion overnight as previously described [12,20]. Primary OA chondrocytes at 80% confluence were used for all the studies described here. Primary OA chondrocytes from different patients were studied for COL2A1, ACAN and COL10A1 expression by qRT-PCR. They showed the chondrogenic phenotype by expressing COL2A1 and ACAN without expression of COL10A1 (data not shown).

### Treatment of chondrocytes with IL-1β and EGCG

OA chondrocytes (1 × 10^6^/ml) were serum starved in Ham's F-12 starving medium for 12 h and were treated with EGCG (100 μM) for 2 h prior to stimulation with IL-1β (5 ng/ml) for different periods of time. OA chondrocytes cultured without IL-1β or EGCG served as controls. Concentration of EGCG was selected on the basis of our previous studies where EGCG (100 μM) showed effective anti-inflammatory effects without affecting cell viability [12-14]. Culture supernatants were used for the antibody array analysis.

### Nitrite assay

NO production was estimated spectrophotometrically by measuring the accumulation of nitrites in the culture supernatants by Griess reaction [21]. Briefly, 100 μl of culture supernatant were mixed with 100 μl of Griess reagent (1% of sulfanilamide in 2.5% H_3_PO_4 _and 0.1% of N-Naphthylethylenediamine dihydrochloride in H_2_O, v/v) for five minutes at room temperature in micro titer plates. The absorbance was measured at 550 nm with microplate reader (Synergy HT, BioTek Instruments Inc., Winooski, VT, USA), and nitrite concentration was calculated using a standard curve prepared with sodium nitrite.

### Protein antibody array

We used a commercial antibody-based array designed to detect 80 different proteins including growth factors, cytokines and chemokines (RayBio Human Cytokine Array V, RayBiotech, Norcross, GA, USA). Experiments were performed essentially as recommended by the manufacturer. Briefly, array membranes were incubated for 30 minutes in 2 ml blocking buffer, then incubated overnight with 1 ml of the culture supernatant and washed. Then, a cocktail of 80 biotinylated antibodies diluted 1:250 was added at 1 ml per array membrane and membranes were kept at 4°C overnight. Membranes were washed and sandwiched antigens were detected by enhanced chemiluminescence by incubating the membranes for two hours with 2 ml of a peroxidase-labeled streptavidin solution (diluted 1:1000) and signals were captured on X-ray films. Arrays that received samples stimulated with either IL-1β or EGCG alone or in combination were processed simultaneously for image acquisition.

### Validation of results

Each spot of the array membrane was quantified by the image analysis software UN-Scan-It (Silk Scientific, Orem, UT, USA) in triplicate with background correction and the mean value was used. The detection of protein spots on microarrays is a semi-quantitative method. Thus normalization can be used to compare the data between arrays (representing different samples) by accounting for the difference in signal intensities on the two arrays as measured by the signals from the positive controls on those arrays. It is important to note that this assay does not measure the absolute concentration of proteins but the normalized signal intensities can be compared to provide measurement of relative concentration in each sample. Positive control has a controlled amount of biotinylated antibody that is attached to the membrane itself and the signal intensity of those spots are dependent on the amount of streptavidin-HRP bound to the antibody and length of exposure time for the detection. Since these factors proportionately affect the absolute signal density of every spot on the array, the relative difference in the positive control signals between arrays will be proportional to the difference in signal between other pairs of corresponding spots on the two arrays. Normalized values were calculated by the formula: X (Ny) = X(y) * P1/P(y). P1 = average signal density of the positive control spot on the reference array. P(y) = average signal density of the positive control spot on array "y". X(y) = signal density for a particular spot on array for sample "y". X (Ny) = normalized value of the particular spot "X" on array for sample "y".

### Enzyme linked immune-sorbant assays (ELISA)

The effect of IL-1β and/or EGCG (100 μM) on the level of IL-6, IL-8 and TNF-α secreted by chondrocytes in the culture medium was further confirmed by ELISAs (R&D Systems, Minneapolis, MN, USA) and all the assays were performed according to the manufacturer's instructions. Limit of detection for IL-6, IL-8 and TNF-α, was > 0.7 pg/ml, 3.5 pg/ml and 1.6 pg/ml respectively.

### NF-κB DNA binding activity assay

Activation of NF-κB p65 in OA chondrocytes was determined using a highly sensitive transcription factor ELISA kit according to the instructions of the manufacturer (Assay Designs, Ann Arbor, MI, USA). This assay uses streptavidin-coated plates with bound NF-κB-biotinylated consensus sequence to capture the active form of NF-κB. The captured active NF-κB is incubated with a specific antibody. The assay was developed with a chemiluminescent substrate and the signal was detected using a luminometer (Lumat LB 9507; Berthold Technologies, Bad Wildbad, Germany), and the values are expressed as relative light units (RLU).

### Quantitative real-time-PCR (RT-PCR)

Real time quantitative-PCR was used to quantify the gene expression of mRNAs for IL-6, IL-8 and TNF-α and other genes with expression of GAPDH used as endogenous control. Total RNA was isolated from OA chondrocytes by TRIzol reagent (Invitrogen, Carlsbad, CA, USA) according to the manufacturer's instruction. First-strand cDNA was synthesized using 1 μg of total RNA and the SuperScript First-Strand cDNA synthesis kit (Invitrogen). PCR amplification was carried out using specific primers (Additional file Table 2), the core kit for SYBR Green (Quanta Biosciences, Gaithersburg, MD, USA) and the Step One Real-Time PCR System (Applied Biosystems, Foster City, CA, USA). Typical profile times used were initial step, 95°C for 10 minutes followed by a second step at 95°C for 15 s and 60°C for 30 s for 40 cycles with melting curve analysis. The level of target mRNA was normalized to the level of GAPDH and compared with control. Data were analyzed using ΔΔCT method [22].

### Western blotting and densitometric analysis

IL-1β-stimulated and control OA chondrocytes were washed with cold PBS and lysed using the cell lysis buffer (50 mM Tris:HCl, pH 7.4; 150 mM NaCl; 1% Triton X-100;0.1% SDS; 0.5% sodium deoxycholate; 1 mM EDTA; 1 mM EGTA; Complete^® ^protease and phosphatase inhibitors) as previously described [23,24]. Total lysate protein (40 μg/lane) was resolved by SDS-PAGE (10% resolving gel with 4% stacking) and transferred to nitrocellulose membranes (BioRad, Hercules, CA, USA). Membranes were blocked with blocking buffer containing non-fat dry milk powder in Tris buffered saline with 0.1% Tween-20 (TBS-T), and probed with 1:500 and 1:1,000 diluted primary antibodies (1:500 to 1:1,000) specific for the target proteins. Immunoreactive proteins were visualized by using 1:5,000 diluted HRP-linked secondary antibodies and enhanced chemiluminescence (GE Health Care, Milwaukee, WI, USA). Bands were scanned and the analysis was performed using Un-Scan-It software. Each band was scanned three times and data were normalized to loading control and expressed as mean ± SD followed by appropriate statistical analysis.

### Statistical analysis

All experiments were performed in duplicate and repeated three times using independent samples. Values shown are mean ± SE unless stated otherwise. Comparisons were performed using Origin 8.1 software package (OriginLab Corporation, Northampton, MA, USA) (one-paired two-tailed *t*-test with one way ANOVA and Tukey's *post-hoc *analysis) and *P *< 0.05 was considered significant.

## Results

### Nitrite level in IL-1β stimulated OA chondrocytes and effects of EGCG

Previously, we have shown that EGCG is an effective inhibitor of IL-1β induced nitric oxide (NO) production in chondrocytes [12] and the concentration of EGCG used (100 μM) was not cytotoxic in 24-hour cultures [13]. To ensure that subsequent protein microarray data provided an accurate representation of the effect of EGCG and IL-1β on chondrocytes, nitrite levels were used as surrogate for NO expression induced by IL-1β and the effect of EGCG on NO expression to verify that the chondrocytes responded reproducibly to both the molecules. As shown in Additional file 1a, nitrite levels significantly increased (*P *< 0.05) upon IL-1β (5 ng/ml) stimulation of OA chondrocytes for 24 hours. Pretreatment of OA chondrocytes with EGCG significantly inhibited (*P *< 0.05) the IL-1β-induced production of NO in human OA chondrocytes confirming the previously reported results.

### Effect of EGCG alone on OA chondrocytes

**Figure 1:**
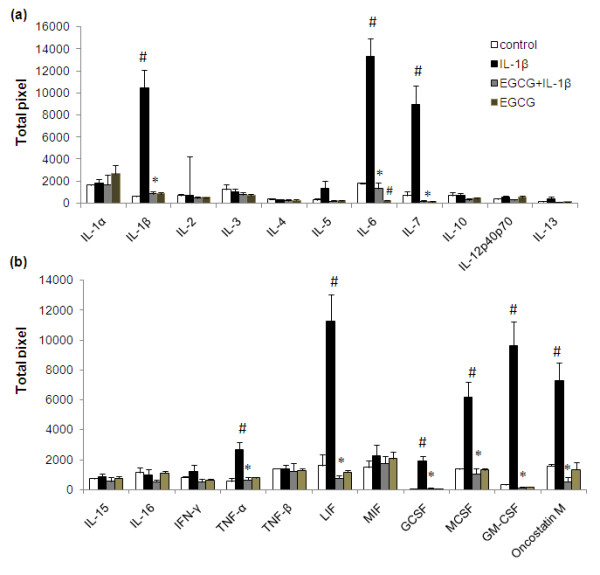
**Inhibition of IL-1β induced cytokine expression in human OA chondrocytes by EGCG (a, b)**. Chondrocytes were pretreated for 2 h with EGCG (100 μM) and then stimulated or not stimulated with IL-1β (5 ng/ml) for 24 h. Values represent Mean ± SE of three different patients. # represents *P *< 0.05 Vs control; * *P *< 0.05 Vs IL-1β stimulated chondrocytes. EGCG, epigallocatechin-3-gallate; GCSF, granulocyte colony stimulating factor; GM-CSF, granulocyte-macrophages colony stimulating factor; IFN-γ, interferon-γ; IL, interleukin; LIF, leukemia inhibitory factor; MCSF, macrophages colony stimulating factor; MIF, macrophage migratory inhibitory factor; TNF, tumor necrosis factor.

**Figure 2:**
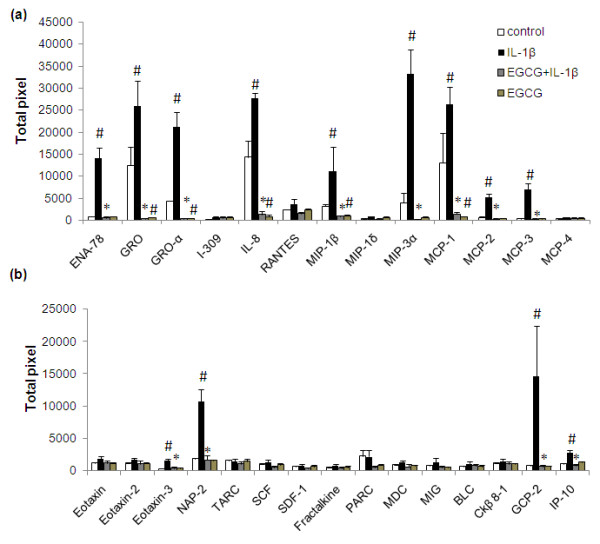
**Effect of EGCG on IL-1β stimulated secretion of chemokines in human OA chondrocytes (a-b)**. Chondrocytes were pretreated for 2 h with EGCG (100 μM) and then stimulated or not stimulated with IL-1β (5 ng/ml) for 24 h. Values represent Mean ± SE of three different patients. # represents *P *< 0.05 Vs control; * *P *< 0.05 Vs IL-1β stimulated chondrocytes. BLC, B-lymphocyte chemoattractant; EGCG, epigallocatechin-3-gallate; ENA-78, epithelial neutrophil activating peptide-78; GCP-2, granulocyte chemotactic protein-2; GRO, growth-related oncogene; IL- interleukin; MCP, monocyte chemotactic protein; MDC, macrophage-derived chemokine; MIG, monokine induced by interferon-gamma; MIP, macrophage inflammatory protein; NAP-2, nucleosome assembly protein-2; RANTES, regulated upon activation normal T-cell expressed and secreted;IP-10,10 kDa interferon gamma-induced protein.

**Figure 3:**
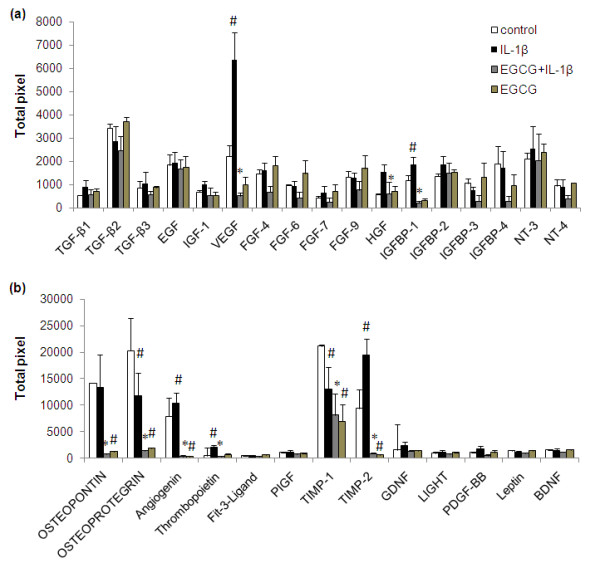
**Effect of EGCG on secretion of growth and angiogenic factors in human OA chondrocytes stimulated with IL-1β (a, b)**. Chondrocytes were pretreated for 2 h with EGCG (100 μM) and then stimulated or not stimulated with IL-1β (5 ng/ml) for 24 h. Values represent Mean ± SE of three different patients. # represents *P *< 0.05 Vs control; * *P *< 0.05 Vs IL-1β stimulated chondrocytes. BDNF, brain-derived neurotrophic factor; EGCG, epigallocatechin-3-gallate; EGF, endothelial growth factor; FGF, fibroblast growth factor; GDNF, glial cell derived neurotrophic factor; HGF, hepatocyte growth factor; IGF, insulin-like growth factor; IGFBP, insulin-like growth factor binding protein; NT, neurotrophin; PDGF-BB, platelet-derived growth factor receptor, beta polypeptide subunit B; PIGF, phosphatidylinositol glycan anchor biosynthesis; TGF, transforming growth factor; TIMP, tissue inhibitor of MMPs; VEGF, vascular endothelial growth factor.

Of the 80 proteins present on the antibody array, treatment with EGCG alone showed no significant change (*P *< 0.05) in the expression of 69 proteins (approximately 86%) relative to control chondrocytes. However, expression of 11 proteins (approximately 14%; *P *< 0.05) was found to be altered upon EGCG treatment. Representative antibody array data, showing effects of EGCG alone on the expression of different proteins in OA chondrocytes are shown in Additional file 1b, e). For the array layout and identification of cytokines please see Additional file 2. The constitutive expression of IL-6 in OA chondrocytes was significantly down regulated (Figure 1; *P *< 0.05) by EGCG treatment, which also significantly reduced (*P *< 0.05) the basal level of some chemokines (Figure 2) such as growth related oncogene (GRO), GRO-α, monocyte chemoattractant protein-1 (MCP-1/CCL2), interleukin-8 (IL-8/CXCL-8), and macrophage inflammatory protein-1β (MIP-1β/CCL4). EGCG also significantly reduced the constitutive production of angiogenin, osteoprotegrin and osteopontin compared to control chondrocytes (Figure 3; *P *< 0.05). OA chondrocytes also constitutively expressed the TIMP-1 and TIMP-2 proteins but their expression was significantly inhibited by EGCG (Figure 3; *P *< 0.05).

### Effect of IL-1β on the expression of inflammatory and catabolic proteins in OA chondrocytes

Treatment of OA chondrocytes with IL-1β resulted in the altered expression of 29 proteins (approximately 36%; Additional file 1b, c, *P *< 0.05). Several specific observations should be mentioned here: (i) chondrocytes stimulated with IL-1β (5 ng/ml) showed a several fold increase in the expression of granulocyte-macrophages colony stimulating factor (GM-CSF), G-CSF, macrophages colony stimulating factor (M-CSF), interleukin-6 (IL-6), IL-7, oncostatin M and leukemia inhibitory factor (LIF) (Figure 1; *P *< 0.05). A slight increase was also observed in the expression of tumor necrosis factor-α (TNF-α) compared to un-stimulated chondrocytes (Figure 1; *P *< 0.05). (ii) High level expression of some chemokines was also actively induced (Figure 2; *P *< 0.05) by IL-1β including epithelial neutrophil activating peptide-78 (ENA-78/CXCL5), GRO, GRO-α, MCP-1, IL-8, MIP-1β, MIP-3α, nucleosome assembly protein-2 (NAP-2/CXCL7) and granulocyte chemotactic protein-2 (GCP-2/CXCL6) in OA chondrocytes. IL-1β-stimulation of OA chondrocytes also showed significant up-regulation of MCP-2(CCL8), MCP-3 (CCL7), IP-10 (CXCL-10) and Eotaxin 3, while the expression of I-309, RANTES and MCP-4 was not significantly induced by IL-1β-stimulation of OA chondrocytes (Figure 2; *P *< 0.05). (iii) Some proteins known to be involved in extracellular matrix remodeling including tissue inhibitors of MMPs (TIMPs), growth and angiogenic factors were also significantly up-regulated by IL-1β and this includes vascular endothelial growth factor (VEGF), IGFBP-1, thrombopoietin, angiogenin, and TIMP-2 (Figure 3; *P *< 0.05). In contrast, a decrease or no effect on the expression of proteins associated with matrix remodelling including osteoprotegrin and TIMP-1 was observed upon IL-1β-stimulation of OA chondrocytes (Figure 3; *P *< 0.05).

### Effect of EGCG on IL-1β-induced protein expression in OA chondrocytes

Additional file 1c, d showed the comparative expression pattern of proteins between IL-1β stimulated chondrocytes in the presence and absence of EGCG. Treatment of OA chondrocytes with EGCG prior to stimulation with IL-1β proved to be surprisingly potent and a broad spectrum inhibitor of IL-1β-induced expression of different cytokines and other proteins in human OA chondrocytes. Overall, relative to IL-1β-stimulated chondrocytes where 28 proteins were significantly up-regulated, treatment with EGCG down regulated the expression of all the up-regulated proteins (Figures 1, 2 and 3). Pretreatment of OA chondrocytes with EGCG, markedly reduced the IL-1β-induced production of GM-CSF, MC-SF, LIF, IL-6, IL-7 and oncostatin M (Figure 1; *P *< 0.05). Pretreatment with EGCG also significantly reduced the expression of TNF-α, and G-CSF in OA chondrocytes stimulated with IL-1β (Figure 1; *P *< 0.05). Results of antibody array showed increased IL-1β in IL-1 stimulated chondrocytes, but EGCG pretreatment significantly inhibited (*P *< 0.05) the IL-1β levels in culture supernatants (Additional file 1e). EGCG pretreatment of OA chondrocytes also showed noticeable inhibitory effects on the production of ENA-78, GRO, GRO-α, IL-8, MIP-1β, MIP-3α, MCP-1, GCP-2, NAP-2 and moderate inhibitory effect on MCP-2 and 3, eotaxin 3 and IP-10 production (Figure 2; *P *< 0.05). Interestingly, EGCG also significantly inhibited the level of angiogenin, thrombopoeitin, VEGF, TIMP-1 and 2, IGFBP-1 in IL-1-stimulated OA chondrocytes (Figure 3; *P *< 0.05). Although, IL-1β significantly down-regulated the expression of osteoprotegrin, EGCG alone also seems to reduce its constitutive production in OA chondrocytes (Figure 3; *P *< 0.05).

### Effect of EGCG on the expression and production of IL-6, IL-8 and TNF-α in chondrocytes

**Figure 4:**
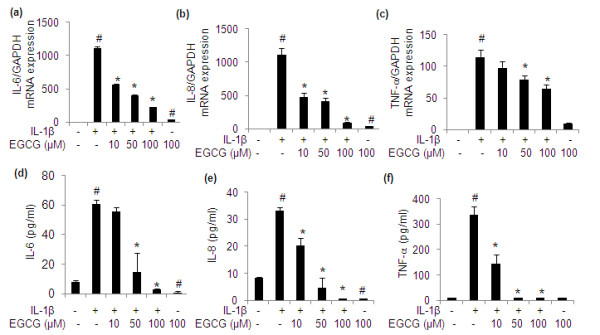
**Effect of EGCG IL-6, IL-8 and TNF-α gene expression and production in IL-1β-stimulated human OA chondrocytes**. Chondrocytes were pretreated with EGCG (10 to 100 μM) for 2 h and stimulated with IL-1β for **(a, b) **8 h or **(c, d, e**, and **f) **24 h. Gene expression for IL-6, IL-8 and TNF-α was determined by quantitative RT-PCR normalized to GAPDH and then compared with the levels present in un-stimulated chondrocytes. Levels of IL-6, IL-8 and TNF-α in the culture supernatants were quantified by sandwich ELISA. Value represents Mean ± SE of three different patients. # represents *P *< 0.05 Vs control; * *P *< 0.05 Vs IL-1β stimulated chondrocytes. EGCG, epigallocatechin-3-gallate; IL-interleukin; TNF, tumor necrosis factor.

Based on the data obtained by antibody arrays, we selected IL-6, IL-8 and TNF-α expression for further confirmation as these proteins have pathophysiological relevance to OA severity and progression. Chondrocytes were pretreated with EGCG (10 to 100 μM) for 2 h and then stimulated with IL-1β for 8 h for mRNA expression (except for TNF-α mRNA which was done at 24 h) and 24 h for protein production. As shown in Figure-4a, pretreatment with EGCG (10 to 100 μM) dose dependently inhibited the IL-1β-induced mRNA expression of IL-6 (*P *< 0.05). To determine whether this inhibition of gene expression also influenced the production of IL-6 protein culture supernatants were assayed using IL-6-specific ELISA. Pretreatment with EGCG (10 to 100 μM) also significantly inhibited the IL-6 production in culture supernatants of stimulated chondrocytes (Figure 4d; *P *< 0.05). Additionally, EGCG pretreatment significantly reduced the basal level of IL-6 production by OA chondrocytes (Figure 4d; *P *< 0.05).

Our results showed that chondrocytes treated with IL-1β had significantly higher expression of IL-8 mRNA similar to protein microarray results, in comparison to un-stimulated chondrocytes (Figure 4b; *P *< 0.05). However, IL-8 mRNA expression showed a significant decline in chondrocytes pretreated with different concentration of EGCG (Figure 4b; *P *< 0.05). When culture supernatants were analyzed for IL-8 protein levels results were similar to those obtained with IL-8 mRNA expression. (Figure 4e; *P *< 0.05). In agreement with the protein array results EGCG also significantly reduced the basal level of IL-8 (Figure 4e; *P *< 0.05). Pretreatment of chondrocytes with EGCG (50 and 100 μM) also significantly inhibited the IL-1β induced mRNA expression of TNF-α compared to IL-1β-stimulated OA chondrocytes (Figure 4c; *P *< 0.05). Pretreatment with EGCG showed a dose dependent decrease in the level of TNF-α protein production in IL-1β-stimulated OA chondrocytes (Figure 4f; *P *< 0.05).

### Role of nuclear factor-kappaB (NF-κB) and mitogen activated protein kinase (MAPKs) pathways in EGCG mediated inhibition of IL-6, IL-8 and TNF-α in IL-1β-stimulated OA chondrocytes

**Figure 5:**
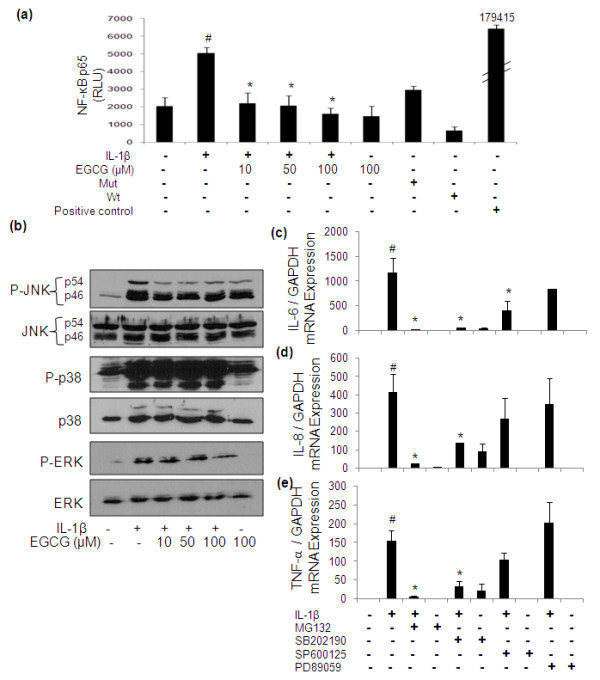
**EGCG mediated inhibition of NF-κB and MAPKs in IL-1β stimulated human OA chondrocytes and regulation of IL-6, IL-8 and TNF-α**. **(a) **Human OA chondrocytes were pretreated with EGCG (10 to 100 μM) for 2 h and stimulated with IL-1β for 24 h. NF-κBp65 was determined in cell lysate by highly sensitive and specific ELISA (Assay design). TNF-α-treated extract of HeLa cells (supplied with the kit) was used as a positive control. The assay is developed with a chemiluminescent substrate and the signal is detected using luminometer (Lumat LB 9507; Berthold Technologies). NF-κB p65 activity was expressed as relative light unit (RLU). **(b) **After pretreatment with EGCG (10 to 100 μM) for one hour, chondrocytes were stimulated with IL-1β for one half-hour, and then phosphorylation of JNK, ERK and p38-MAPK was determined by Western immunobloting. **(c, d**, and **e) **Effect of specific inhibitors for mitogen activated protein kinases and NF-κB on the gene expression of IL-6, IL-8 and TNF-α in IL-1β stimulated human OA chondrocytes. Primary chondrocytes were pretreated with specific inhibitors for 2 h and were stimulated with IL-1β for 6 h. Relative gene expression of IL-6, IL-8 and TNF-α normalized to GAPDH and compared with un-stimulated control, were determined by quantitative RT-PCR. Concentrations of specific inhibitors of p38 (SB202190), JNK (SP600125), ERK (PD98059) and NF-κB (MG132) used in these studies were 100 μM, 10 μM, 50 μM and 100 μM, respectively. Value represents Mean ± SE of three different patients. # represents *P *< 0.05 Vs control; * *P *< 0.05 Vs IL-1β stimulated chondrocytes. EGCG, epigallocatechin-3-gallate;IL-interleukin; ERK, extracellular signal-regulated kinases; JNK, cJun-N-termial Kinases; MAPKs, mitogen activated protein kinases; NF-κB, nuclear factor kappa-B; TNF, tumor necrosis factor.

NF-κB is an important transcriptional regulator of inflammatory gene expression and plays a crucial role in immune and inflammatory response. However, activation of MAPKs is also intimately associated with the expression of pro-inflammatory genes. As shown in Figure 5a, EGCG significantly (*P *< 0.05) inhibited the IL-1β-induced activation of NF-κB in OA chondrocytes as previously reported [12,25]. As IL-6, IL-8 and TNF-α genes are NF-κB dependent; its inhibition by EGCG provides an explanation for the inhibition of IL-6, IL-8 and TNF-α expression in OA chondrocytes. EGCG also inhibited the activation of JNK in OA chondrocytes (Figure 5b) [13]; we also studied the role of MAPKs in IL-1β-induced expression of IL-6, IL-8 and TNF-α in OA chondrocytes. We used the pharmacological agents, MG132 (NF-κB inhibitor), SB202190 (p38-MAPK inhibitor), SP600125 (JNK-MAPK inhibitor) and PD98059 (ERK inhibitor) to dissect the role of individual MAPKs in the expression of IL-6, IL-8 and TNF-α in OA chondrocytes. Treatment of OA chondrocytes with the proteasome inhibitor MG132, blocked the IL-1β-induced IL-6, IL-8 and TNF-α expression as determined by quantitative real-time PCR (Figures 5c, d and 5e; *P *< 0.05). When the role of individual MAPKs inhibition and expression of IL-6, IL-8 and TNF-α in OA chondrocytes was studied, we found that the activation of p38-MAPK was essential for the optimal expression of IL-6, IL-8 and TNF-α as inhibition of p38-MAPK nearly obliterated their expression in IL-1β-treated OA chondrocytes (Figures 5 c, d and 5e). Inhibition of JNK activation in OA chondrocytes also down-regulated the IL-1β-induced expression of IL-6 but the effect was not as dramatic as was obtained with p38 MAPK inhibition (Figure 5c; *P *< 0.05). Inhibition of ERK activation showed virtually no inhibition of IL-6, IL-8 and TNF-α gene expression in IL-1β-treated OA chondrocytes indicating that ERK activation is not essential for their expression. Thus, inhibitory effects of EGCG on IL-6, IL-8 and TNF-α expression may be related to the inhibition of NF-κB and JNK activation in IL-1β-stimulated OA chondrocytes. These results also demonstrate that inhibition of IL-6, IL-8 and TNF-α can also be targeted via inhibition of p38-MAPK without inhibiting NF-κB.

### Effect of EGCG on the expression of TRAF-6 and IRAK-1 in IL-1β-stimulated OA chondrocytes

**Figure 6:**
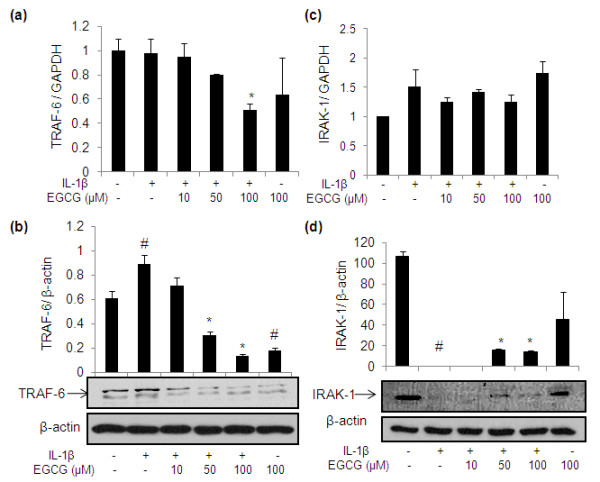
**Gene expression and production of TRAF-6 and IRAK-1 in IL-1β-stimulated human OA chondrocytes**. **(a) **and **(c) **Effect of EGCG pretreatment on the gene expression of TRAF-6 and IRAK-1 in IL-1β-stimulated OA chondrocytes, respectively. **(b) **and **(d) **Effect of EGCG on the production of TRAF-6 and IRAK-1 in IL-1β-stimulated OA chondrocytes, respectively. Value represents Mean ± SD of three different patients. # represents *P *< 0.05 Vs control; * *P *< 0.05 Vs IL-1β stimulated chondrocytes. EGCG, epigallocatechin-3-gallate; IL, interleukin; IRAK-1, interleukin-1 receptor-associated kinase 1; TRAF-6, TNF receptor-associated factor 6.

TRAF-6 and IRAK-1 are important signaling intermediates in transducing signals to the cell after the binding IL-1 to IL-1R on the cell surface. OA chondrocytes were pretreated with EGCG (10 to 100 μM) for one hour and then stimulated with IL-1β for one half-hour for both mRNA expression and protein production. Treatment with IL-1β caused significant increase in TRAF-6 protein expression, whereas pre-incubation with 50 and 100 μM concentration of EGCG appeared to inhibit TRAF-6 protein expression significantly, which is consistent with the significant inhibition of TRAF-6 mRNA expression observed in OA chondrocytes pre-treated with EGCG (Figure 6a, b; *P *< 0.05). EGCG pretreatment did not show any significant change on IRAK-1 mRNA expression in OA chondrocytes stimulated with IL-1β (Figure 6c). Treatment with IL-1β in OA chondrocytes completely degraded the IRAK-1 protein compared to un-stimulated OA chondrocytes (Figure 6d; *P *< 0.05), whereas pre-incubation with 50 and 100 μM concentrations of EGCG inhibited the IL-1β-induced degradation of IRAK-1 (Figure 6d).

### Real-time PCR verification

**Figure 7:**
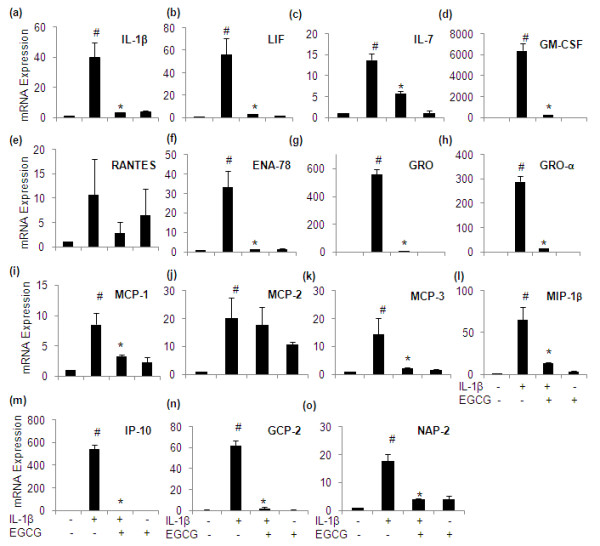
**Real-time PCR verification of the expression of IL-1β**. **(a)**, LIF **(b)**, IL-7 **(c)**, GM-CSF **(d)**, RANTES **(e)**, ENA-78 **(f)**, GRO **(g)**, GRO-α **(h)**, MCP-1 **(i)**, MCP-2 **(j)**, MCP-3 **(k)**, MIP-1β **(l)**, IP-10 **(m)**, GCP-2 **(n)**, NAP-2 **(o)**. Value represents Mean ± SD of three different patients. # represents *P *< 0.05 Vs control; * *P *< 0.05 Vs IL-1β stimulated chondrocytes. ENA-78, epithelial neutrophil activating peptide-78; GCP-2, granulocyte chemotactic protein-2; GM-CSF, granulocyte-macrophages colony stimulating factor; GRO, growth-related oncogene; IL-interleukin; IP-10,10 kDa interferon gamma-induced protein; LIF, leukemia inhibitory factor; MCP, monocyte chemotactic protein; MIP, macrophage inflammatory protein; NAP-2, nucleosome assembly protein-2; RANTES, regulated upon activation normal T-cell expressed and secreted.

In order to confirm the effects of EGCG obtained by protein array analysis, the expression of 15 additional genes was also analyzed by real-time PCR (Figure 7a-o). The primer sequences for the genes verified by RT-PCR are given in Additional file 3. This analysis demonstrated a significant inhibitory effect of EGCG on the expression pattern of all the genes analyzed (Figure 7; *P *< 0.05) except MCP-2 (Figure 7j), which in contrast with the array results was not found to be down regulated by EGCG in IL-1β-stimulated OA chondrocytes (Figure 2; *P *< 0.05). Among the genes whose expression was verified by real-time PCR analysis, EGCG appeared to inhibit the expression of GM-CSF, GRO, GRO-α and IP-10 maximally in IL-1β-stimulated OA chondrocytes in agreement with the results shown above.

### Role of NF-κB pathways in EGCG mediated inhibition of verified genes in IL-1β-stimulated OA chondrocytes

**Figure 8:**
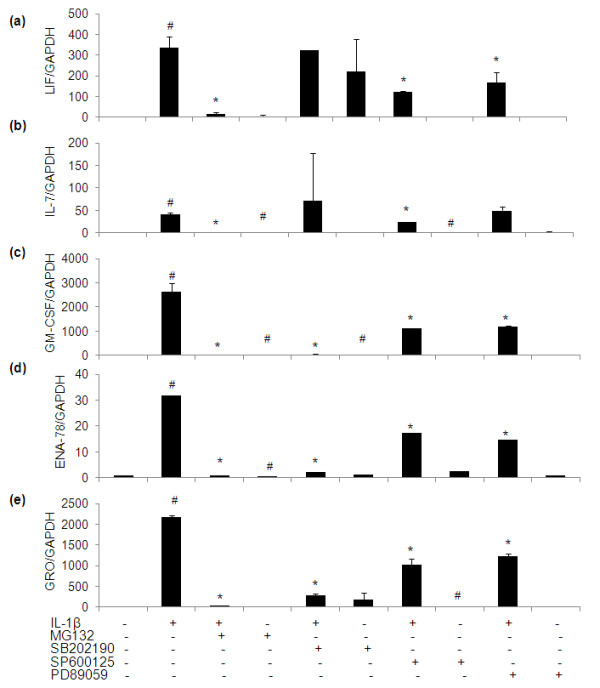
**Effect of specific inhibitors of MAPKs and NF-κB pathways on the gene expression of pro-inflammatory cytokines and chemokines in IL-1β-stimulated human OA chondrocytes**. Primary chondrocytes were pretreated with specific inhibitors for 2 h and were stimulated with IL-1β for 6 h. Relative gene expression of LIF **(a)**, IL-7 **(b)**, GM-CSF **(c)**, ENA-78 **(d) **and GRO **(e) **was normalized to GAPDH and compared with un-stimulated control, were determined by quantitative RT-PCR. Concentrations of specific inhibitors of p38 (SB202190), JNK (SP600125), ERK (PD98059) and NF-κB (MG132) used in these studies were 100 μM, 10 μM, 50 μM and 100 μM, respectively. Value represents Mean ± SD of three different patients. # represents *P *< 0.05 Vs control; * *P *< 0.05 Vs IL-1β stimulated chondrocytes. ENA-78, epithelial neutrophil activating peptide-78; GM-CSF, granulocyte-macrophages colony stimulating factor; GRO, growth-related oncogene; IL-interleukin; LIF, leukemia inhibitory factor; MAPKs, mitogen activated protein kinases; NF-κB, nuclear factor kappa-B.

**Figure 9:**
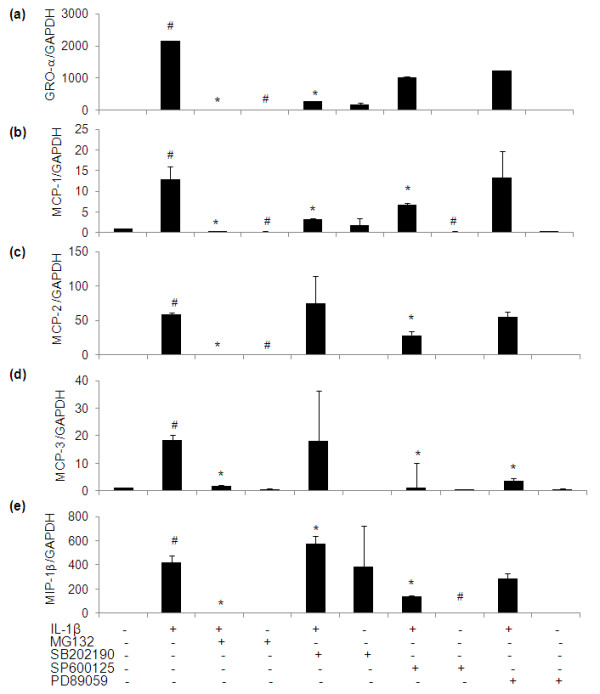
**Effect of specific inhibitors of MAPKs and NF-κB pathways on the gene expression of pro-inflammatory chemokines in IL-1β-stimulated human OA chondrocytes**. Primary chondrocytes were pretreated with specific inhibitors for 2 h and were stimulated with IL-1β for 6 h. Relative gene expression of GRO-α **(a)**, MCP-1 **(b)**, MCP-2 **(c)**, MCP-3 **(d) **and MIP-1β **(e) **was normalized to GAPDH and compared with un-stimulated control, were determined by quantitative RT-PCR. Concentrations of specific inhibitors of p38 (SB202190), JNK (SP600125), ERK (PD98059) and NF-κB (MG132) used in these studies were 100 μM, 10 μM, 50 μM and 100 μM, respectively. Value represents Mean ± SD of three different patients. # represents *P *< 0.05 Vs control; * *P *< 0.05 Vs IL-1β stimulated chondrocytes. GRO, growth-related oncogene; MAPKs, mitogen activated protein kinases; MCP, monocyte chemotactic protein; MIP, macrophage inflammatory protein; NF-κB, nuclear factor kappa-B.

**Figure 10:**
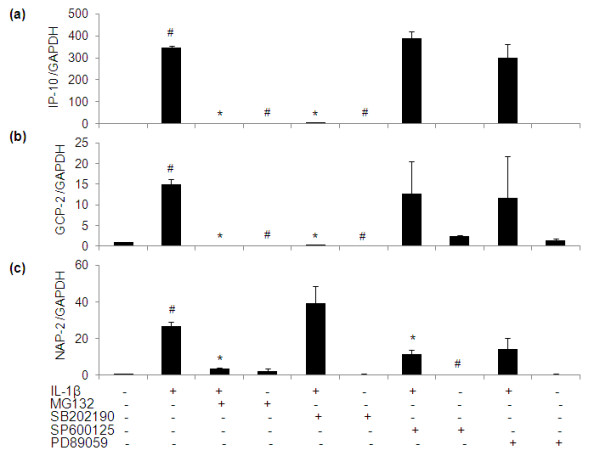
**Effect of specific inhibitors of MAPKs and NF-κB pathways on the gene expression of pro-inflammatory cytokines and chemokines in IL-1β-stimulated human OA chondrocytes**. Primary chondrocytes were pretreated with specific inhibitors for 2 h and were stimulated with IL-1β for 6 h. Relative gene expression of IP-10 **(a)**, GCP-2 **(b) **and NAP-2 **(c) **was normalized to GAPDH and compared with un-stimulated control, were determined by quantitative RT-PCR. Concentrations of specific inhibitors of p38 (SB202190), JNK (SP600125), ERK (PD98059) and NF-κB (MG132) used in these studies were 100 μM, 10 μM, 50 μM and 100 μM, respectively. Value represents Mean ± SD of three different patients. # represents *P *< 0.05 Vs control; * *P *< 0.05 Vs IL-1β stimulated chondrocytes. GCP-2, granulocyte chemotactic protein-2; IP-10, 10 kDa interferon gamma-induced protein; MAPKs, mitogen activated protein kinases; NAP-2, nucleosome assembly protein-2; NF-κB, nuclear factor kappa-B.

A large body of direct and indirect evidence links the NF-κB pathway to the inflammation and cartilage degeneration in OA. Thus, inhibition of NF-κB holds promise in regulating IL-1β-induced inflammatory response in human OA chondrocytes [3,26]. We also studied the effect of NF-κB inhibitor (MG132) on the expression of LIF, IL-7, GM-CSF, ENA-78, GRO, GRO-α, MCP-1, MCP-2, MCP-3, MIP-1β, IP-10, GCP-2, and NAP-2. As shown in Figures 8, 9 and 10, stimulation with IL-1β significantly increased the mRNA expression levels of all of these genes (*P *< 0.05). Interestingly, blockage of NF-κB activation in IL-1β-stimulated OA chondrocytes caused a significant decrease in the expression of all of them (*P *< 0.05). Further, inhibition of NF-κB activation in OA chondrocytes treated with MG132, but not stimulated with IL-1β, also caused a significant decrease in the constitutive expression of IL-7, GM-CSF, ENA-78, GRO-α, MCP-1, MCP-2, IP-10 and GCP-2 (*P *< 0.05). As EGCG is an effective inhibitor of NF-κB (Figure 5a; *P *< 0.05) [12], the inhibitory effects of EGCG on the expression of these genes in IL-1β-stimulated OA chondrocytes could be due to the inhibition of NF-κB activation.

### Role of MAPKs in regulation of gene expression and their modulation by EGCG in IL-1β-stimulated OA chondrocytes

We have previously shown that EGCG inhibits JNK phosphorylation (Figure 5b) in IL-1β-stimulated OA chondrocytes but has no effect on p38- or ERK-MAPKs activation [13]. Here we examined the role of MAPKs in the regulation of the newly identified genes induced by IL-1β in OA chondrocytes and the possible role of EGCG in their modulation via MAPKs. As shown in Figures 8, 9 and 10, IL-1β significantly increased the constitutive expression levels of all of these genes in OA chondrocytes (*P *< 0.05) in agreement with the array results. When the effect of MAPKs inhibition on the regulation of LIF and MCP-3 was studied in IL-1β-treated OA chondrocytes, inhibition of JNK-and ERK-MAPK (SP600125 and PD98059, respectively) was found to be necessary for a significant down-regulation of IL-1β-induced LIF and MCP-3 expression (*P *< 0.05). While inhibition of JNK alone resulted in significant down-regulation of IL-1β-induced IL-7, MIP-1β, MCP-2 and NAP-2 mRNA expression in OA chondrocytes (*P *< 0.05), inhibtion of p38-MAPK alone (SB202190) was sufficient to inhibit IL-1β-induced expression of GRO-α, IP-10 and GCP-2 mRNA significantly (*P *< 0.05) in OA chondrocytes. Treatment of OA chondrocytes with IL-1β and p38- and JNK-MAPK inhibitors significantly inhibited the mRNA expression of MCP-1 (*P *< 0.05). The expression of ENA-78, GM-CSF and GRO was found to be regulated by p38, JNK and ERK-MAPKs (*P *< 0.05) in IL-1β-stimulated OA chondrocytes.

## Discussion

EGCG, a bioactive component of green tea has been shown to possess anti-cancer and anti-inflammatory activity with a potential for anti-arthritic effects and is of considerable interest in this regard [27]. Many cytokines have been implicated in causing synovial tissue activation and damage to cartilage and subchondral bone in OA [28]. The most widely investigated cytokine in OA is IL-1β, because it strongly induces cartilage catabolic agents [3,6], and inhibits proteoglycan and collagen synthesis [29]. In this study, a spectrum of biological effects of EGCG are described for the first time that provide evidence of modulation of cartilage protective and inflammation suppressing events by EGCG in IL-1β-stimulated OA chondrocytes. In addition, several new genes were found to be induced by IL-1β in OA chondrocytes and their modulation by EGCG was studied.

We used the larger screening capacity of the array technology to analyze for changes in cytokines levels induced by IL-1β in OA chondrocytes, and to search for their possible modulation by EGCG. Our test system had limitations in that the screening was only limited to 80 cytokines (broadly defined) and some mediators with pathophysiological relevance to OA may have been neglected. However, the protein array technology is easy to use and supported by the specificity of protein recognition by antibodies. Moreover, we used nitrite production as an indicator of chondrocytes activation by IL-1β and its suppression by EGCG [12], to demonstrate that the concentrations of IL-1β and EGCG used were sufficient to induce and detect the change in cellular response. Unlike the dramatic response of IL-1β alone, pre-incubation of chondrocytes with EGCG rendered the chondrocytes essentially unresponsive to subsequent IL-1β stimulation, and thereby EGCG appeared to be highly chondroprotective.

Our results showed the increased IL-1β levels in IL-1β stimulated OA chondrocytes, but EGCG pretreatment inhibited the IL-1β mRNA and protein levels in OA chondrocytes. These results suggest that there may be a direct inhibitory effect of EGCG on IL-1β expression. This is supported by a recent study showing that EGCG (50 mg/L) inhibited the expression of IL-1β mRNA and reduced the IL-1β level at cutaneous wound sites in a mouse model [30]. Adaptor proteins involved in IL-1 signaling such as IRAK and TRAF-6, may also constitute important therapeutic targets for OA. Phophorylated IRAK becomes associated with TRAF6, which is a downstream transducer required for NF-κB activation [31]. Our results showed that treatment with IL-1β caused a significant increase in TRAF-6 protein, whereas pre-incubation with EGCG inhibited the mRNA and protein expression of TRAF-6. Autophosphorylation of IRAK promotes its dissociation from this complex, which is followed by its polyubiquitination, and subsequent degradation by the 26S proteosome system [32,12]. Our results showed that IL-1β-stimulation caused complete degradation of IRAK compared to control, whereas pre-incubation with EGCG inhibited IRAK degradation in OA chondrocytes. This is important because our study identifies the EGCG mediated inhibition of IRAK degradation, a crucial event in the IL-1β-activated signal transduction pathway, in OA chondrocytes may be related to its anti-inflammatory activity as this may inhibit the signal transduction.

Studies have reported the presence of IL-1β in diseased areas along with staining of TNF, IL-6 and IL-8 [33,34]. Evidence to date indicates that in addition to IL-1, IL-6, TNF-α and IL-8 can also promote articular cartilage extracellular matrix degradation or synergize with other cytokines to amplify and accelerate cartilage destruction [3-6]. Our array data showed that IL-1β-stimulation of OA chondrocytes significantly induced the mRNA as well as protein expression of IL-6, IL-8 and TNF-α, which was significantly down-regulated by EGCG. Using highly sensitive ELISAs, we confirmed the array data which showed the significant dose dependent inhibitory effect of EGCG on the IL-1β-induced production of IL-6, IL-8 and TNF-α in OA chondrocytes. This is supported by the previous study where EGCG was shown to inhibit IL-6 production in RA-synovial fibroblasts [35]. The pro-inflammatory pathways play central roles in the degradation of OA cartilage. IL-1β activates multiple phosphorylation-dependent signaling pathways including NF-κB and MAPKs (that is, extracellular signal regulated kinase (ERK), c-Jun N-terminal kinase (JNK) and p38-MAPKs), which lead to the coordinated expression of many genes that encode cytokines, chemokines, and other mediators involved in synthesis and further amplification of the inflammatory reaction [36,37]. We studied the involvement of NF-κB and MAPKs in IL-1β mediated inflammatory response and their regulation by EGCG. Our data showed that EGCG inhibited the NF-κB activation and JNK-activation in IL-1β-stimulated OA chondrocytes as reported previously [12,13]. Data obtained from NF-κB inhibitor (MG132) in the presence and absence of IL-1β revealed the role of NF-κB in the regulation of IL-6, IL-8 and TNF-α in OA chondrocytes. Recent studies have suggested that the inhibition of NF-κB activation down-regulates TNF-α expression in AGE-BSA stimulated OA chondrocytes [16]. In the present study, EGCG was also found to be a potent inhibitor of the IL-1β-induced expression of IL-6 via inhibition of JNK-MAPK activation. However, modulatory effects of EGCG on the IL-8 and TNF-α production appeared to be specifically via inhibition of NF-κB activation, as no role of JNK pathway was observed in the regulation of IL-8 and TNF-α expression using a specific inhibitor of JNK (SP600125) in IL-1β-stimulated OA chondrocytes. Inhibition of p38-MAPK (SB202190) was found to potently suppress the expression of IL-6, IL-8 and TNF-α in IL-1β-stimulated OA chondrocytes independent of NF-κB inhibition. Taken together these results suggest that activation of p38-MAPK is responsible for the optimum expression of IL-6, IL-8 and TNF-α in IL-1β-stimulated OA chondrocytes, while JNK appeared to be critical for the expression of IL-6 but not for IL-8 and TNF-α in IL-1β-stimulated OA chondrocytes.

LIF is a cytokine whose expression is found to be high in OA cartilage and can be enhanced by IL-1β stimulation [38,39]. Interestingly, LIF is a potent suppressor of chondrocyte proteoglycan synthesis [40], induces collagenase 3 (MMP-13) [41], IL-1 and IL-6 expression [42] and up-regulates COX-2 and PGE_2 _synthesis [43]. LIF also stimulates proteoglycan resorption and its inhibitors are considered to have a therapeutic potential for the treatment of RA [44]. To our knowledge, this report is the first demonstrating that EGCG significantly inhibits LIF mRNA and protein expression in IL-1β-stimulated OA chondrocytes. Thus, EGCG may be developed as a safe and effective inhibitor of LIF in OA. Colony stimulating factors (CSF) were among the first cytokines found in RA synovial fluid [45], and their increased expression was correlated with the severity of rheumatoid arthritis (RA) [46] and recently with OA pathogenesis [47]. There are numerous reports of GM-CSF precipitating or exacerbating established inflammatory disorders [48]. Moreover, antagonism of GM-CSF markedly reduces established disease in mouse models of RA and has a comparable effect to that of anti-TNF treatment [49]. Another new gene demonstrated in this report to be over-expressed in IL-1β-stimulated OA chondrocytes was IL-7. Our results demonstrate for the first time that EGCG inhibits IL-1β-induced mRNA and protein expression of IL-7 and GMCSF in OA chondrocytes. IL-7 is considered as a potential contributor to OA pathogenesis [50]. Although very little progress has been made in developing and testing anti-IL-7 therapy in animal models of OA, there is an increased interest in IL-7 as a potent stimulator of matrix metalloproteinase expression in OA [50,51]. In this study, we have also investigated the role of NF-κB and MAPKs on the EGCG mediated inhibition of LIF, GM-CSF and IL-7 in IL-1β-stimulated chondrocytes. Our study showed that EGCG inhibits the expression of LIF, GM-CSF and IL-7 via inhibition of NF-κB in IL-1β-stimulated OA chondrocytes. Our results also point out that inhibition of JNK using specific inhibitor (SP600125) in the presence of IL-1β down-regulates LIF and IL-7 mRNA expression, suggesting the inhibition of JNK activation as an important event in the EGCG mediated down regulation of LIF and IL-7 expression in these cells. In this study it was also discovered that GM-CSF expression was regulated by JNK, ERK and p38-MAPKs in OA chondrocytes in response to IL-1β. As EGCG did not inhibit ERK and p38-MAPK activation in IL-1β-stimulated OA chondrocytes, EGCG mediated down-regulation of GM-CSF expression may be mediated via NF-κB or by JNK-MAPK inhibition.

Chemokines are important regulators of chronic inflammation, typically orchestrating leucocyte migration and expression of chemokine receptor has been demonstrated in chondrocytes [52] and synovial cells [53]. They have been implicated in the pathophysiology of inflammatory diseases including OA [54,55]. Our data demonstrated that ENA-78, GRO, GRO-α, MCP-1 and 3, MIP-1β, MIP-3α, GCP-2 and NAP-2 were expressed and released into the medium upon stimulation with IL-1β. Our data also showed that their induction and expression were suppressed by EGCG in IL-1β- stimulated OA chondrocytes. Secretion of IP-10 was also up-regulated by IL-1β and inhibited by EGCG. EGCG alone treatment was also effective in down-regulating the basal level of expression of GRO, GRO-α, MCP-1 and MIP-1β in OA chondrocytes. Consistent with the present findings, induction of chemokines has been previously described after stimulation of chondrocytes with IL-1 [56,57], but their suppression by EGCG has not been previously described in OA chondrocytes. However, Ahmed *et al. *(2006) reported similar inhibitory effects of EGCG (50 μM) on RANTES, ENA-78, GRO-α, and MCP-1 production in IL-1β-stimulated RA-synovial fibroblasts [58]. These results indicated that the chemokine blockade by EGCG might be a promising novel therapeutic strategy for RA and OA. Recently, administration of EGCG markedly diminished the severity of collagen induced arthritis, macrophage infiltration, and the amount of CCL2-synthesizing osteoblasts [59]. Further, EGCG has been reported to decrease MCP-1 mRNA and protein expression and its receptor CCR2 expression in THP-1 cells [60]. Chemokine signaling pathway molecules may be up-regulated in arthritis by mechanical injury [52]. In accordance with the results of other group, where they showed the role of NF-κB in EGCG mediated down regulation of chemokine production [58], down-regulation of ENA-78, GRO, GRO-α, MCP-1 and 3, MIP-1β, MIP-3α, GCP-2 and NAP-2 by EGCG in the present study may also be mediated through the inhibition of NF-κB. Suppression of MAPKs activation has also been linked to anti-inflammatory activity; we, therefore, tested whether EGCG mediates its inhibitory effects on chemokine expression via inhibition of MAPKs and the role of this pathway in the regulation of these selected chemokines. In the present study, p38-specific, JNK-specific and ERK-specific inhibitors (SB202190, SP600125, and PD98059) reduced the expression of ENA-78, and GRO. However, the expression of GRO-α, IP-10 and GCP-2 was susceptible only to inhibition of p38-MAPK activation. Expression of MIP-1β and MCP-1 was dependent on the activation of p38- and JNK MAPKs while expression of MCP-3 was found to be regulated by the JNK and ERK-MAPKs. The expression level of NAP-2 was only affected by JNK inhibition in IL-1β-stimulated OA chondrocytes.

## Conclusions

In summary, our study demonstrates that EGCG exerts differential inhibitory expression of inflammatory mediators in articular chondrocytes in the presence of IL-1β. These inhibitory effects are mediated via inhibition of MAPKs and NF-κB pathways in OA chondrocytes. Our results also demonstrate that some chemokines require the activation of p38-MAPK for optimum expression. The use of protein antibody array allowed the identification of several new target proteins of EGCG in OA chondrocytes. This provides novel insights into the anti-inflammatory potential of EGCG on IL-1β-induced inflammatory response. Although, EGCG did not show direct stimulatory effect on the proteins associated with the cartilage anabolic response, it was found to be a surprisingly broad spectrum inhibitor of IL-1β-induced cartilage catabolic and inflammatory factors known to be associated with the pathogenesis of arthritis. These results support the development and use of EGCG as an anti-inflammatory/anti-arthritic agent for prevention/treatment of OA.

## Abbreviations

ACAN: aggrecan; ADAMTS: a disintegrin and metalloproteinase with thrombospondin motifs; AP-1: activator protein-1; COX-2: cyclooxygenase-2; EGCG: epigallocatechin-3-gallate; ENA-78: epithelial neutrophil activating peptide-78: ERK: extracellular signal-regulated kinases; GCP-2: granulocyte chemotactic protein-2; GM-CSF: granulocyte-macrophages colony stimulating factor; GRO: growth-related oncogene; IGFBP-1: insulin-like growth factor-binding protein 1; IL-1β: interleukin-1β; IP-10: interferon-gamma-inducible protein-10; IRAK-1: interleukin-1 receptor-associated kinase 1; IRB: Institutional Review Board; JNK: cJun-N-termial kinases; LIF: leukemia inhibitory factor; MAPK: mitogen activated protein kinases; MAPKs: mitogen activated protein kinases; MCP-1: monocyte chemotactic protein-1; M-CSF: macrophages colony stimulating factor; MIP-1β: macrophage inflammatory protein-1beta; MMP: matrix metalloproteinases; NAP-2: nucleosome assembly protein-2; NF-κB: nuclear factor kappa-B; NO: nitric oxide; OA: osteoarthritis; PGE_2: _prostaglandin E_2_; RA: rheumatoid arthritis; RANTES: regulated upon activation normal T-cell expressed and secreted; RLU: relative light units; TIMP-1: tissue inhibitor of MMPs-1; TNF-α: tumor necrosis factor-alpha; TRAF-6: TNF receptor-associated factor 6; VEGF: vascular endothelial growth factor.

## Competing interests

The authors declare that they have no competing interests.

## Authors' contributions

NA carried out the experimental work, collection, interpretation and manuscript drafting. TMH conceived of the study and its design, coordinated the study, interpreted data and drafted the manuscript. All authors have read and approved the final manuscript.

## Supplementary Material

Additional file 1 **Effect of EGCG on nitrite production by human OA chondrocytes stimulated with IL-1β**. **(a) **Chondrocytes were pretreated with EGCG (100 μM) for 2 h then stimulated with IL-1β (5 ng/ml) for 24 h and the culture supernatant was used for providing the cytokine antibody array. Bar represents mean ± SD of three patients, # represents *P *< 0.05 Vs control; * *P *< 0.05 Vs IL-1β stimulated chondrocytes. **(b-e) **Represents cytokine-antibody arrays showing the effects of EGCG on the expression pattern of cytokines produced by human OA chondrocytes upon IL-1β-stimulation. For the array layout and identification of cytokines please see additional file 2. Click here for file

Additional file 2 **Comprehensive map of cytokines antibody array**. Click here for file

Additional file 3 **Primer sequences used to quantify gene expression with real-time PCR**. Click here for file
