# Supplementary figures and images for: Epigallocatechin-3-gallate suppresses the global interleukin-1beta-induced inflammatory response in human chondrocytes
Source: Arthritis Res Ther. 2011 Jun 17;13(3):R93. doi: 10.1186/ar3368 (PMC3218908; doi:10.1186/ar3368)

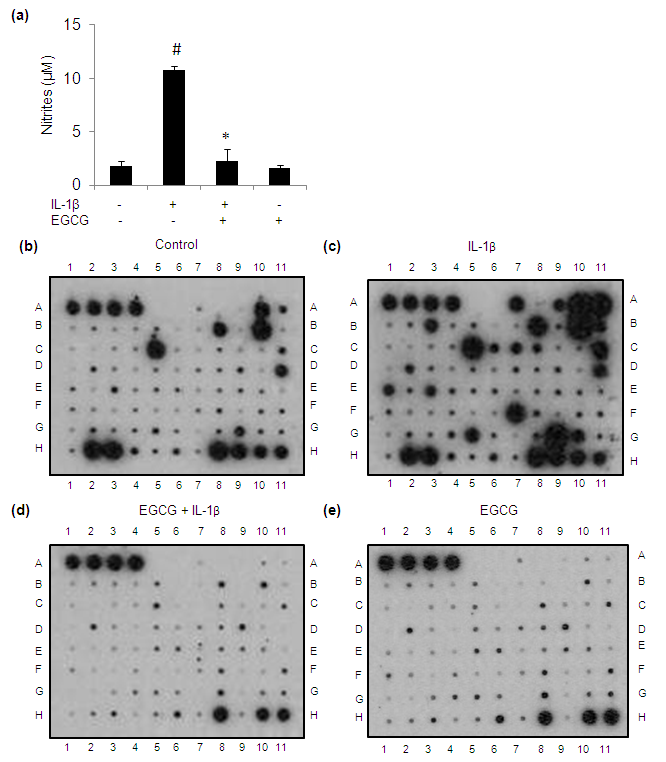

Supplement: Additional file 1 — Effect of EGCG on nitrite production by human OA chondrocytes stimulated with IL-1β. (a) Chondrocytes were pretreated with EGCG (100 μM) for 2 h then stimulated with IL-1β (5 ng/ml) for 24 h and the culture supernatant was used for providing the cytokine antibody array. Bar represents mean ± SD of three patients, # represents P < 0.05 Vs control; * P < 0.05 Vs IL-1β stimulated chondrocytes. (b-e) Represents cytokine-antibody arrays showing the effects of EGCG on the expression pattern of cytokines produced by human OA chondrocytes upon IL-1β-stimulation. For the array layout and identification of cytokines please see additional file 2. [file ar3368-S1.TIFF]
