# Supplementary material for: Epigallocatechin-3-gallate suppresses the global interleukin-1beta-induced inflammatory response in human chondrocytes
Source: Arthritis Res Ther. 2011 Jun 17;13(3):R93. doi: 10.1186/ar3368 (PMC3218908; doi:10.1186/ar3368)
Supplement: Additional file 2 — Comprehensive map of cytokines antibody array. [file ar3368-S2.DOC]

Comprehensive map of cytokines antibody array.

|  | **1** | **2** | **3** | **4** | **5** | **6** | **7** | **8** | **9** | **10** | **11** |
| --- | --- | --- | --- | --- | --- | --- | --- | --- | --- | --- | --- |
| **A** | POS | POS | POS | POS | Neg | Neg | ENA-78 | GC-SF | GM-CSF | GRO | GRO-α |
| **B** | I-309 | IL-1α | IL-1β | IL-2 | IL-3 | IL-4 | IL-5 | IL-6 | IL-7 | IL-8 | IL-10 |
| **C** | IL-12p40p70 | IL-13 | IL-15 | IFN-γ | MCP-1 | MCP-2 | MCP-3 | MC-SF | MDC | MIG | MIP-1β |
| **D** | MIP-1δ | RANTES | SCF | SDF-1 | TARC | TGF-β1 | TNF-α | TNF-β | EGF | IGF-1 | Angiogenin |
| **E** | Oncostatin M | Thrombopoietin | VEGF | PDGF-BB | Leptin | BDNF | BLC | Ckβ 8-1 | Eotaxin | Eotaxin-2 | Eotaxin-3 |
| **F** | FGF-4 | FGF-6 | FGF-7 | FGF-9 | Fit-3ligand | Fractalkine | GCP-2 | GDNF | HGF | IGFBP-1 | IGFBP-2 |
| **G** | IGFBP-3 | IGFBP-4 | IL-16 | IP-10 | LIF | LIGHT | MCP-4 | MIF | MIP-3α | NAP-2 | NT-3 |
| **H** | NT-4 | Osteopontin | Osteoproteg-erine | PARC | PIGF | TGF-β 2 | TGF-β 3 | TIMP-1 | TIMP-2 | POS | POS |
