# Supplementary material for: Epigallocatechin-3-gallate suppresses the global interleukin-1beta-induced inflammatory response in human chondrocytes
Source: Arthritis Res Ther. 2011 Jun 17;13(3):R93. doi: 10.1186/ar3368 (PMC3218908; doi:10.1186/ar3368)
Supplement: Additional file 3 — Primer sequences used to quantify gene expression with real-time PCR. [file ar3368-S3.DOC]

Primer sequences used to quantify gene expression with real-time PCR.

| Gene name | Gene ID | Primer Sequence (5’ to 3’) |
| --- | --- | --- |
| glyceraldehyde-3-phosphate dehydrogenase (GAPDH) | NM_002046 | F, 5’-TCG ACA GTC AGC CGC ATC TTC TTT-3’  R, 5’-ACC AAA TCC GTT GAC TCC GAC CTT-3’ |
| Interleukin-6 (IL-6) | NM_000600 | F, 5’-AAA TTC GGT ACA TCC TCG ACG GCA-3’  R, 5’-AGT GCC TCT TTG CTG CTT TCA CAC-3’ |
| Interleukin-8 (IL-8) | NM_000584 | F, 5’-AGA AAC CAC CGG AAG GAA CCA TCT-3’  R, 5’-AGA GCT GCA GAA ATC AGG AAG GCT-3’ |
| Interleukin-1β (IL-1β) | NM_000576 | F, 5’-AAA CAG ATG AAG TGC TCC TT-3’  R, 5’-TGG AGA ACA CCA CTT GTT GC-3’ |
| Interleukin-7 (IL-7) | NM_000880 | F, 5’-GTG GCT TCC GTG CAC ACA TTA ACA -3’  R, 5’-CCA GCT GGC CTG AAT CAA AGC AAT-3’ |
| Tumor necrosis factor-α (TNF-α) | NM_000595 | F, 5’-AGG ACG AAC ATC CAA CCT TCC CAA-3’  R, 5’-TTT GAG CCA GAA GAG GTT GAG GGT-3’ |
| Leukemia Inhibitory factor (LIF) | NM_002309 | F, 5’-ACG AGG ATG TGG CTG TTG AGA TGT -3’  R, 5’-ATG AAG CAG GAA GGA GAA GGC AGT -3’ |
| granulocyte-macrophages colony stimulating factor (GM-CSF) | NM_000758 | F, 5’-AAA TGT TTG ACC TCC AGG AGC CGA -3’  R, 5’-AGG TGA TAA TCT GGG TTG CAC AGG -3’ |
| chemokine (C-C motif) ligand 5 (RANTES) | NM_002985 | F, 5’-GAA GGA AGT CAG CAT GCC TC -3’  R, 5’-AGC CGA TTT TTC ATG TTT GC -3’ |
| epithelial neutrophil activating peptide-78 (ENA-78) | NM_002994 | F, 5’-ATC CTC CAA TCT TCG CTC CT-3’  R, 5’-GCT GGA CAG GAG GCT CAT AG-3’ |
| growth related oncogene (GRO) | NM_002089 | F, 5’-GAA AGC TTG CCT CAA TCC TG -3’  R, 5’-CAC CAG TGA GCT TCC TCC TC-3’ |
| growth related oncogene-α (GRO-α) | NM_001511 | F, 5’-CTC AAG AAT GGG CAG AAA GC-3’  R, 5’-TCA GGA ACA GCC ACC AAT AA -3’ |
| monocyte chemoattractant protein-1 (MCP-1) | NM_002982 | F, 5’-TCG CTC AGC CAG ATG CAA TCA ATG -3’  R, 5’-TGG AAT CCT GAA CCC ACT TCT GCT -3’ |
| monocyte chemoattractant protein-2 (MCP-2) | NM_005623 | F, 5’-GAA GCT TCC TCG CAA CTT TGT GGT -3’  R, 5’-GGA TCA GCA CAG ACT TGC TTG CTT -3’ |
| monocyte chemoattractant protein- 3 (MCP-3) | NM_006273 | F, 5’-AAA TCC CTA AGC AGA GGC TGG AGA -3’  R, 5’-AAA GTC CTG GAC CCA CTT CTG TGT -3’ |
| macrophage inflammatory protein-1β (MIP-1β) | NM_002984 | F, 5’-GAA GCT TCC TCG CAA CTT TGT GGT -3’  R, 5’-GGA TCA GCA CAG ACT TGC TTG CTT -3’ |
| chemokine (C-X-C motif) ligand 10 (IP-10) | NM_001565 | F, 5’-AAC CTC CAG TCT CAG CAC CAT GAA -3’  R, 5’-ACT AAT GCT GAT GCA GGT ACA GCG -3’ |
| granulocyte chemotactic protein-2 (GCP-2) | NM_002993 | F, 5’-GAC AGA GCT GCG TTG CAC TTG TTT -3’  R, 5’-AAC TTG CTT CCC GTT CTT CAG GGA -3’ |
| nucleosome assembly protein-2 (NAP-2) | NM_005969 | F, 5’-ACA CAG AAG TGG GTC CAG GAC TTT -3’  R, 5’-AAC CAC TCT GAG AAA GGA CAG GGT -3’ |
